# Inversion of Hyperpolarized ^13^C NMR Signals through Cross-Correlated Cross-Relaxation in Dissolution DNP Experiments

**DOI:** 10.1021/acs.jpcb.2c03375

**Published:** 2022-06-08

**Authors:** Mattia Negroni, David Guarin, Kateryna Che, Ludovica M. Epasto, Ertan Turhan, Albina Selimović, Fanny Kozak, Samuel Cousin, Daniel Abergel, Geoffrey Bodenhausen, Dennis Kurzbach

**Affiliations:** † Faculty of Chemistry, Institute of Biological Chemistry, University Vienna, Währinger Str. 38, 1090 Vienna, Austria; ‡ Athinoula A. Martinos Center for Biomedical Imaging, Department of Radiology, Massachusetts General Hospital, Charlestown, Massachusetts 02129, United States; § Polarize ApS, 1808 Frederiksberg, Denmark; ∥ Institut de Chimie Radicalaire—UMR 7273, Saint-Jérôme Campus, Av. Esc. Normandie Niemen, Aix-Marseille Université/CNRS, 13397 Marseille Cedex 20, France; ⊥ Laboratoire des Biomolécules, LBM, Département de chimie, École Normale Supérieure, PSL University, Sorbonne Université, CNRS, 24 rue Lhomond, 75005 Paris, France

## Abstract

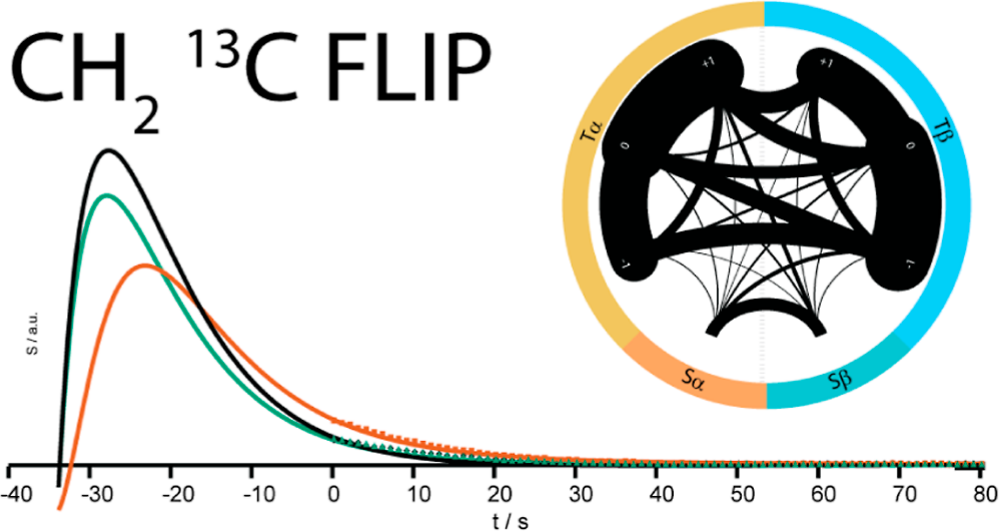

Dissolution dynamic nuclear polarization (DDNP) is a versatile tool to boost signal amplitudes in solution-state nuclear magnetic resonance (NMR) spectroscopy. For DDNP, nuclei are spin-hyperpolarized “*ex situ*” in a dedicated DNP device and then transferred to an NMR spectrometer for detection. Dramatic signal enhancements can be achieved, enabling shorter acquisition times, real-time monitoring of fast reactions, and reduced sample concentrations. Here, we show how the sample transfer in DDNP experiments can affect NMR spectra through cross-correlated cross-relaxation (CCR), especially in the case of low-field passages. Such processes can selectively invert signals of ^13^C spins in proton-carrying moieties. For their investigations, we use schemes for simultaneous or “parallel” detection of hyperpolarized ^1^H and ^13^C nuclei. We find that ^1^H → ^13^C CCR can invert signals of ^13^C spins if the proton polarization is close to 100%. We deduce that low-field passage in a DDNP experiment, a common occurrence due to the introduction of so-called “ultra-shielded” magnets, accelerates these effects due to field-dependent paramagnetic relaxation enhancements that can influence CCR. The reported effects are demonstrated for various molecules, laboratory layouts, and DDNP systems. As coupled ^13^C–^1^H spin systems are ubiquitous, we expect similar effects to be observed in various DDNP experiments. This might be exploited for selective spectroscopic labeling of hydrocarbons.

## Introduction

Recent years have witnessed an increasing interest in so-called hyperpolarization techniques that enable the detection of nuclear magnetic resonance (NMR) signals with significantly enhanced intensities.^1,2^ Hyperpolarization techniques provide the potential to overcome some of the limitations imposed by the intrinsically weak signals detected in conventional NMR spectroscopy. Signal enhancements of up to 4 orders of magnitude allow one to reduce acquisition times and sample concentrations.

In particular, the development of dissolution dynamic nuclear polarization (DDNP)^3−6^ has significantly stimulated recent uses of hyperpolarized NMR. Applications have been found, for example, NMR of proteins,^7−11^ ligand binding studies,^12−14^ metabolomics,^15−17^ interaction monitoring,^18,19^ NMR of long-lived states,^20−22^ and metabolic imaging.^23−27^ The commercialization of DDNP equipment has further helped popularize this technique.^28^

For DDNP, a target molecule can be hyperpolarized “*ex situ*” in a dedicated apparatus that enables microwave irradiation of paramagnetically doped samples at low temperatures (close to 1 K) and in high magnetic fields (typically >3 T). After the build-up of the hyperpolarization, the sample is dissolved (typically with a burst of superheated D_2_O) and transferred to an NMR spectrometer for NMR detection in the liquid state. One critical criterion that needs to be fulfilled by the target molecules is that longitudinal relaxation times should be long enough to allow for sample transport from the DNP system to the NMR spectrometer—a process that typically takes 1 to 5 s depending on the experimental setup.

The magnetic field along the transfer path of the sample depends critically on the layout of the laboratory.^29−31^ Stray fields of different magnets may not be known accurately. This is particularly true for stray fields near the bore openings of shielded NMR magnets, where the fields may be entirely canceled or even undergo a reversal.

Here, we report unexpected effects occuring during sample transfer on spectra detected in DDNP experiments. We demonstrate how *cross-correlated cross-relaxation* (CCR) between hyperpolarized protons and adjacent carbon-13 nuclei can lead to ^13^C signal inversion. Such effects cannot be observed in conventional thermal equilibrium NMR. However, they dominate NMR spectra when proton polarizations reach values larger than several tens of percent in solution, which is often the case in DDNP experiments. Furthermore, such effects are boosted during low-field transfer passages due to the presence of paramagnetic molecules in the dissolved sample.

We have observed these effects in experiments performed on two entirely different DDNP systems installed in two different laboratories in Vienna and Paris. In addition, we have probed such hyperpolarized CCR effects using two different samples, namely, pyruvate-1–^13^C dissolved in ethylene glycol (EG) and methanol mixed with glycerol-*d*_8_.

## Methods

All DDNP experiments were performed using two-stage setups, where the sample is hyperpolarized in a separate DNP system and then dissolved and transferred to an NMR spectrometer for detection. In all cases, the samples traveled through magnetic tunnels that provided a magnetic field of *B*_Tunnel_ > 0.5 T between the DNP magnet and the NMR spectrometer. In the Vienna setup, both ends of the tunnels^29^ were suspended *ca.* 30 cm above the bores of the polarizer and NMR magnets. Between the polarizer and the entrance to the tunnel, a pulsed solenoidal magnet^32^ has been used in some experiments, providing a field of *B*_Solenoid_ > 0.2 T. Likewise, the space between the exit from the tunnel and the entrance of the bore of the NMR magnet could be bridged by a pulsed solenoid^32^ if desired. The pulsed solenoids ended *ca.* 5 cm before the bore of the magnets. In the Paris setup, the polarizer end of the tunnel was somewhat farther from the magnet bore (∼70 cm), and no additional solenoid was used.

### Sample Preparation

For one sample type, 500 mM ^13^C1-labeled pyruvate and 40 mM TEMPOL were dissolved in a 5:4:1 mixture of EG, D_2_O, and H_2_O. For the second sample type, methanol-OD was mixed 1:1 with glycerol-*d*_8_ and 15 mM TEMPOL.

### Dissolution Dynamic Nuclear Polarization

**Figure 1:**
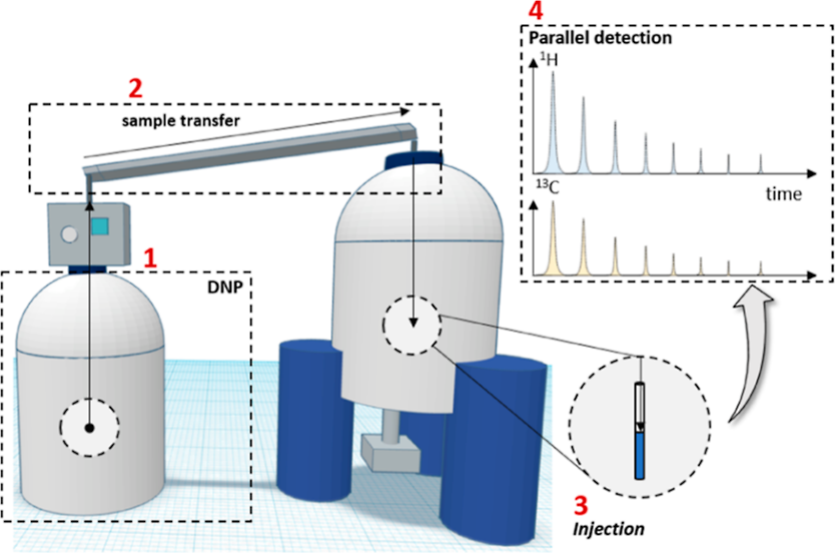
Schematic of DDNP. The samples are hyperpolarized at low temperatures in a dedicated DNP magnet (1) before being (2) dissolved and pneumatically propelled to an NMR spectrometer, where (3) the sample is injected for detection into an NMR tube (4). The hyperpolarization can be detected simultaneously (“in parallel”) on the ^1^H and ^13^C channels using multiplexed receiver technology.

In Vienna, the DDNP experiments were performed as described in ref (32). In brief, 100 μL of a paramagnetically doped methanol/glycerol sample was hyperpolarized for 1 h at 1.4 K in a magnetic field of *B*_0,DNP_ = 6.7 T either positively at a microwave frequency of 188.08 GHz or negatively at a frequency 188.4 GHz. Dissolution was performed with 5 mL of D_2_O at 15.0 bar and 240 °C. The transfer of the dissolved hyperpolarized liquid to the NMR spectrometer took 4 s. The ^1^H and ^13^C signals were detected simultaneously once per second on a Bruker NEO 500 MHz spectrometer equipped with a BBFO Prodigy cryogenic probe head, using 1 and 30° flip angles, respectively. The setup is sketched in Figure 1.

In Paris, DDNP experiments were performed on a Bruker prototype operating at 6.7 T and 1.2 K. Again, 50 μL of a paramagnetically doped sample was used. In contrast to the experiments in Vienna, ^13^C hyperpolarization was boosted by ^1^H–^13^C cross-polarization.^33^ The optimal amplitude of the spin-locking ^13^C radiofrequency field was γ*B*_1_/(2π) = 50 kHz, and the duration of the Hartmann–Hahn contact was 0.4 < τ_SL_ < 0.5 s. A gated microwave field^34^ with a power of 350 mW at 188.4 GHz, modulated with a 100 MHz amplitude sawtooth function with a modulation frequency of 2 kHz, was used to saturate part of the electron paramagnetic resonance spectrum of the free radicals. Dissolution and transfer to the detection NMR spectrometer were performed with 5 mL of D_2_O at 10.5 bar and 180 °C. The transfer of the dissolved hyperpolarized liquid to the NMR spectrometer took 5 s. The ^13^C signals were detected on a Bruker 400 MHz spectrometer using a 10 mm BBO broadband probe at room temperature. Pulses with 10° angles were applied only to ^13^C at intervals of 1 s.

The magnetic fields between the DNP and NMR systems in Vienna were measured using a Hirst GM08 Gauss meter.

### Simulations

All simulations were carried out using the SpinDynamica^35^ Software package for Mathematica (time dependence of the magnetization), RedKite^36^ (relaxation matrices), or a home-written script in MATLAB (field dependence of paramagnetic relaxation rates). All codes and scripts can be downloaded (see the Code Availability section). Multiplet intensities have been fitted using the MATLAB-based “fitnlorentzian.m” function. The most critical simulation codes are furthermore shown explicitly in the Supporting Information.

## Results

We demonstrate the unexpected signal inversion effects for two samples: pyruvate-1–^13^C dissolved in EG and deuterated glycerol mixed with protonated methanol. Although the two systems were studied in different laboratories using different DDNP systems, we observed similar effects. After transfer between DNP and NMR magnets, ^13^C nuclei with neighboring hyperpolarized protons in CH_2_ and CH_3_ moieties display inverted signals.

**Figure 2:**
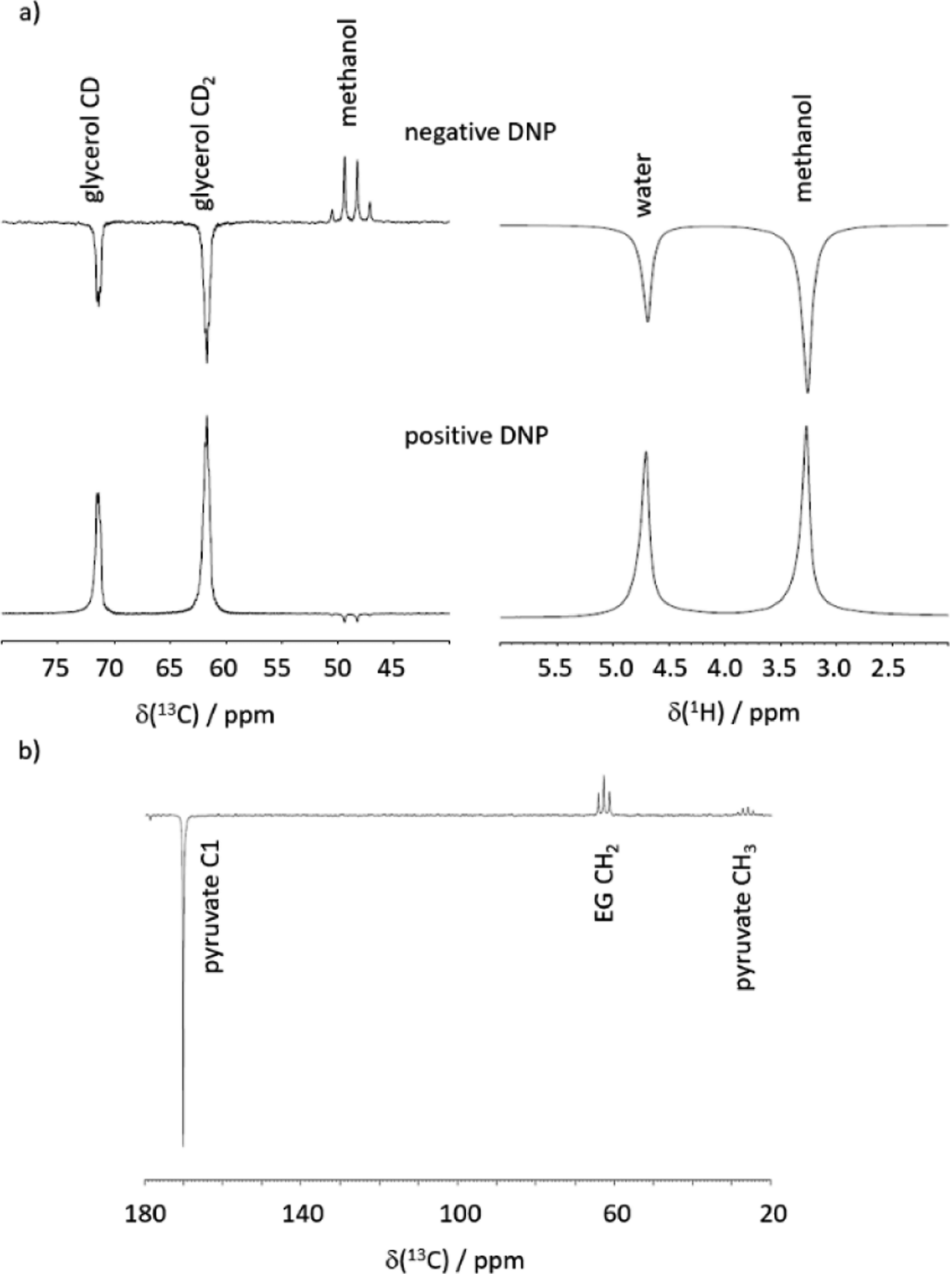
(a) ^1^H and ^13^C spectra of glycerol-*d*_8_ and methanol in natural abundance detected in parallel after negative (top) and positive (bottom) DNP. Note that the methanol ^13^C signal has an inverted sign with respect to that of glycerol-*d*_8_. (b) ^13^C spectra of pyruvate-1–^13^C and EG detected after negative DNP. Note that the EG signals are also inverted with respect to that of pyruvate C1.

Figure 2a shows ^1^H and ^13^C spectra for the methanol/glycerol-*d*_8_ mixture detected in parallel after the dissolution of positively (bottom) or negatively (top) hyperpolarized samples. All ^1^H and ^13^C signals of glycerol-*d*_8_ show positive and negative amplitudes as expected. However, the ^13^C-methanol signals have the opposite amplitudes. The signal enhancements ε for methanol are similar or weaker (ε ≈ −3200 for positive and ε ≈ +400 for negative DNP) than those of glycerol-*d*_8_ (ε ≈ +7600 and −1700, respectively).

Figure 2b displays similar results for the pyruvate/EG mixture. After negative DNP, dissolution, and transfer, the 1–^13^C NMR signal of pyruvate is negative, as expected, while the pronated ^13^CH_2_ signals of EG, as well as those of ^13^CH_3_ in pyruvate, are inverted relative to those of pyruvate C1. Note the intense signal of the methyl group compared to that of C1 despite the selective labeling of the latter.

The signal enhancements obtained using negative DNP for pyruvate-^13^C and EG were ε ≈ −19,000 and +14,000, respectively.

## Theory

To discuss and analyze the signal inversion presented above, this section provides a brief overview of the spin physics underlying the ^13^CH_2_ and ^13^CH_3_ groups of EG and methanol-OD.

### Methylene ^13^CH_2_ Moieties

It is well-known that two interacting degenerate protons generate four stationary nuclear spin states. Three of them constitute triplet states (T). In the Zeeman basis, these can be expressed as
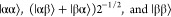
1The fourth state is a singlet state (S), which we express as
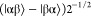
2

Importantly, as pointed out by Levitt and co-workers,^37^ relaxation via dipolar couplings or random field fluctuations between the singlet and triplet manifolds is forbidden within the Redfield approach, which corresponds to the second-order perturbation theory. For protons (as for carbon spins, too), several relaxation mechanisms are active—we here consider chemical shift anisotropy (CSA), dipole–dipole (DD) couplings, CSA–DD cross-terms, and random field relaxation (rnd). However, the dipolar and random field contributions are typically the strongest: if they are neglected in simulations, this significantly prolongs the lifetime of nonequilibrium populations.^20,38,39^

**Figure 3:**
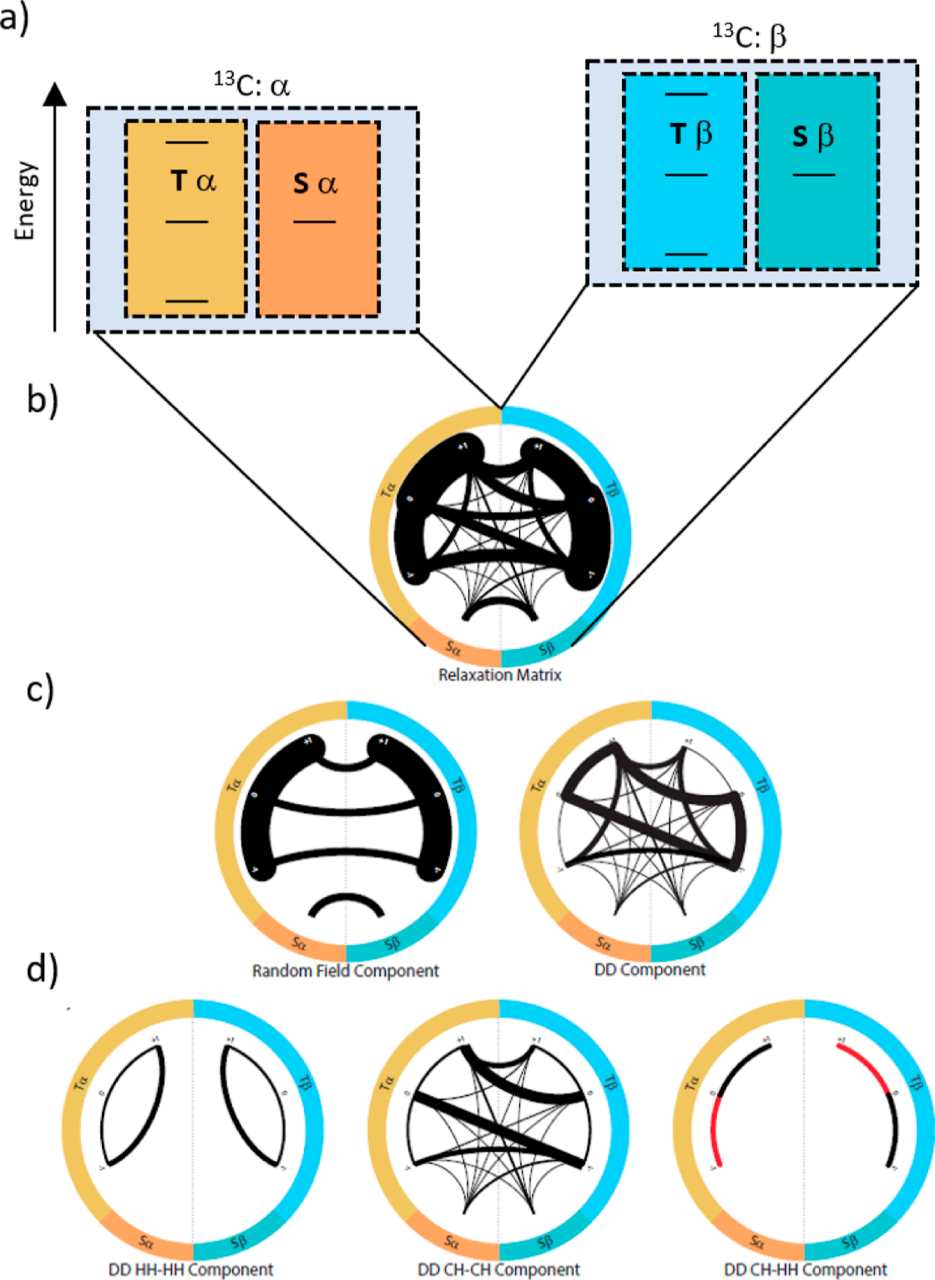
Graphical representation of ^13^CH_2_ spin state levels: (a) spin energy levels. (b) Representation of the relaxation matrix (dipolar plus random field) elements connecting the different spin states. The spin states (energy levels) are represented on the outer ring (T and S states represented in orange and yellow, respectively). The left side corresponds to the α carbon-13 spin state and the right side corresponds to the β state. Black lines connecting the states correspond to possible relaxation pathways. The thicker lines indicate higher rates and faster relaxation. Asymmetric, that is, “diagonal”, relaxation pathways can convert proton polarization into carbon polarization. Hence, it becomes evident which contributions cause the reported signal inversion effect. (c) Representation of the random field (rnd) relaxation matrix. (d) Representation of various components of the DD relaxation matrix. Red indicates negative relaxation rates.

In the presence of a third interacting spin (carbon-13 in a ^13^CH_2_ group), the two manifolds are split into four. Figure 3a shows the resulting energy diagram. Importantly, the third spin enables a weak yet significant population flow between the S and T manifolds due to DD CCR involving the dipoles between the central carbon and the two protons (denoted CH–CH DD in Figure 3).

To help the reader visualize the relaxation pathways, we developed a comprehensive representation of the different states and the flow of populations between them (Figure 3b–d). Note that the graphical representation refers to the relaxation between populations of eigenstates. The different spin states are represented by the outer ring (triplet states on the top and singlet states at the bottom). The ^13^C spin mirrors the S and T states vertically, creating eight levels. The correlation to a more common representation is also shown. Panels 3b–d visualize the relaxation pathways as black lines between the levels; the thicker the line, the higher the relaxation rate. The relaxation rates have been computed using SpinDynamica by expressing the relaxation superoperator through the standard semi-classical treatment of spin relaxation detailed in ref (21) (further details can be found in the Supporting Information).

Three important observations can be made: (i) considering the entire relaxation matrix (Figure 3b), it can be seen that the flow within either the triplet manifold or the singlet manifold is much faster than the flow between them. (ii) Investigating the dipolar and random field relaxation mechanisms independently (Figures 3c,d), we find that the random field contribution can efficiently relax protons and carbon spins. However, the flow between ^13^C states is only possible when the ^1^H state remains unchanged. Hence, proton polarization cannot directly relax into carbon polarization. (iii) DD CCR involving both carbons and protons can efficiently cause relaxation between two different ^13^C states. However, in contrast to random field relaxation, the ^13^C relaxation pathway connects two different ^1^H levels. Hence, proton polarization can be converted into carbon polarization. The difference between points (ii) and (iii) is reflected in Figure 3 through the symmetry with respect to the vertical axis. Random field relaxation leads to a symmetric representation, while DD relaxation entails an asymmetric (diagonal) connection between the proton states on each site. The usefulness of the circular representation becomes evident: only when these representations involve an asymmetric pathway that crosses the central vertical axis can polarization be transferred from a proton to a carbon nucleus or *vice versa*.

It should be noted that the polarization transfer is more efficient (corresponding to thicker lines) within the triplet manifold than between the triplet and singlet manifolds, which is expected, as this process is not symmetry-forbidden. Hence, longitudinal ^1^H_z_ magnetization can be converted into ^13^C_z_ polarization more efficiently than a singlet–triplet population imbalance. Furthermore, note that the ^13^C polarization produced via CCR from ^1^H spins shows an inverted sign due to the CH–CH DD pathway. This is again reflected in the fact that the relaxation pathway is not symmetric with respect to the central axis in Figure 3. In other words, higher energy proton states are converted into lower energy carbon states and *vice versa*. Hence, the polarization transfer involves a change in sign.

### Methyl ^13^CH_3_ Moieties

The situation is more complex for a methyl group as the *C*_3*v*_ symmetry and the additional proton need to be considered. However, a reasoning similar to that for the methylene group applies. The eight ^1^H spins states can be grouped into manifolds of three irreducible representations, that is, A, E_a_, and E_b_. Four states are in the A manifold, two states are in the E_a_ manifold, and the remaining two states are in the E_b_ manifold:A states:
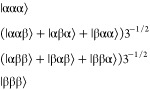
3E_a_ states:
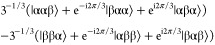
4E_b_ states:
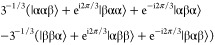
5

**Figure 4:**
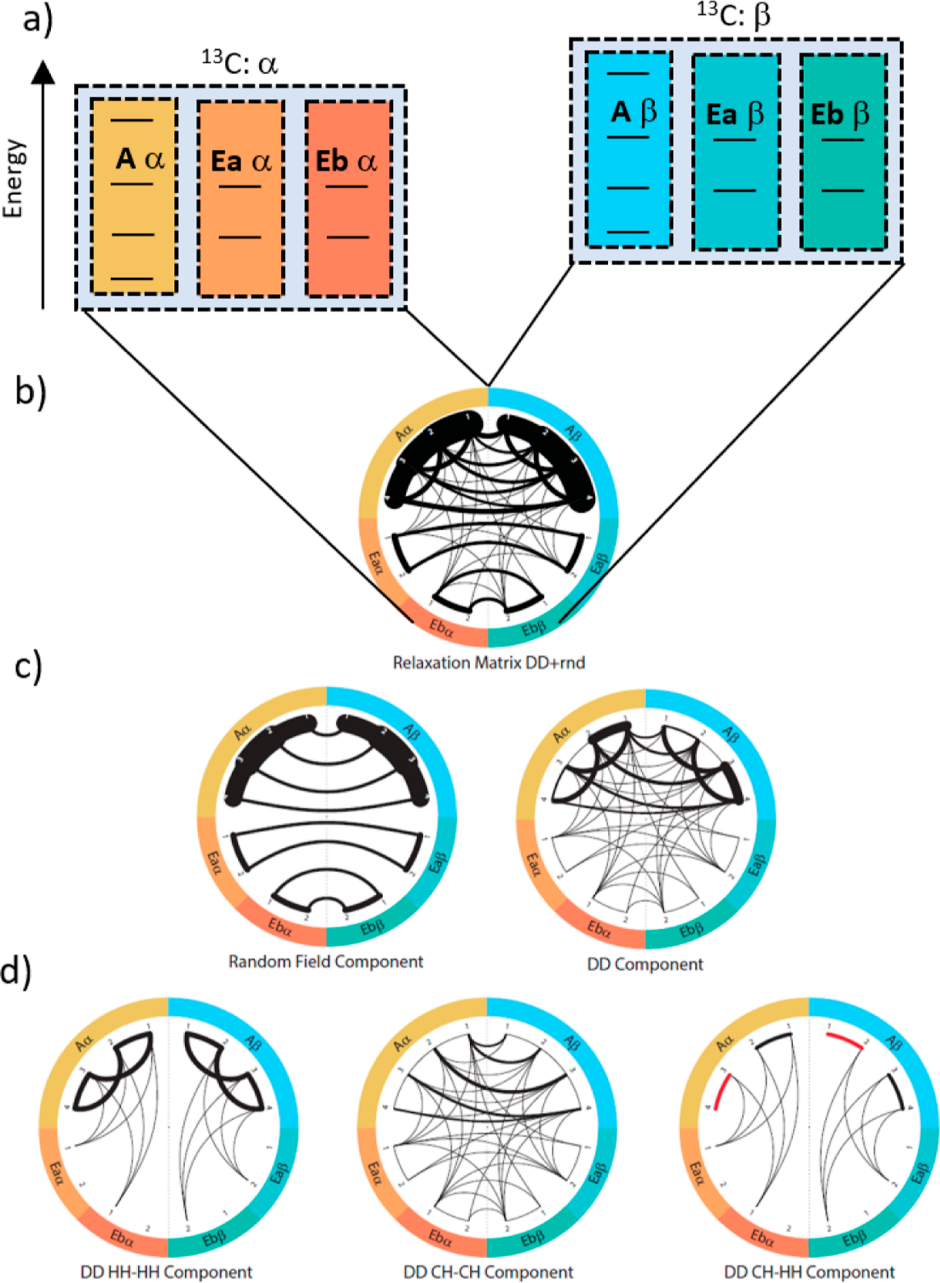
Graphical representation of ^13^CH_3_ spin state levels: (a) spin energy levels. (b) Representation of the relaxation matrix (dipolar plus random field) elements connecting the different spin states. The spin states (energy levels) are represented on the outer ring (A and E states represented in orange and yellow, respectively). The left side corresponds to the α carbon-13 spin state and the right side corresponds to the β state. Black lines connecting the states correspond to possible relaxation pathways. The thicker lines indicate faster relaxation rates. Asymmetric, that is, “diagonal”, relaxation pathways can convert proton polarization into carbon polarization. Hence, it becomes evident which contribution causes the reported signal inversion effect. (c) Representation of the random field relaxation matrix. (d) Representation of the various components of the DD relaxation matrix; red lines indicate negative values.

In addition, we must consider the carbon spin states leading to a total of 16 states for the ^13^CH_3_ moiety. Similar to Figure 3, Figure 4 displays the energy levels (panel a) and the possible relaxation pathways (panels b to d) for the methyl group between the populations of eigenstates. Again, transitions between spin manifolds of different symmetries are forbidden to the first order. Hence, for the methyl group, a long-lived population imbalance between the A and E states can be created when overpopulating either of the manifolds.

Importantly, the relaxation pathway due to the DD CH–CH component is again asymmetric with respect to the vertical symmetry axis in Figure 4. Hence, as explained above, this pathway causes a conversion of proton polarization into carbon polarization of the opposite sign. Another similarity to the CH_2_ case is that the relaxation of the A–E spin state imbalance does not produce ^13^C_z_ magnetization as efficiently as longitudinal ^1^H magnetization.

The only marked difference between the methylene and methyl cases is that the HH–HH and CH–HH DD CCR components can cause a transition between the A and E manifolds, while no such transition between T and S can be observed in methylene groups. The internal methyl group rotation must be infinitely fast to establish a “perfect” separation between the irreducible representations,^21^ which is not the case in a typical methyl group.

Note that the representations in Figures 3 and 4 are incomplete as they only show relaxation between populations without considering possible coherences. A detailed analysis of our simulations has revealed how these components, even if present, do not produce a critical contribution to the overall relaxation. Therefore, we decided to omit them in the graphical visualization. Furthermore, the matrix elements with values below 1% of the highest relaxation contribution are omitted for clarity.

## Discussion

The data in Figure 2 demonstrate that the protonated ^13^C nuclei of methanol, pyruvate, and EG are strongly affected during sample transport, as attested by the fact that their signals have opposite signs. In contrast, the deuterated ^13^CD_2_ nuclei in glycerol-*d*_8_, as well as the quaternary ^13^C1 signal of pyruvate, display the expected results, that is, strong signal enhancements with signs that correspond to the polarization built up via DNP before dissolution. In the following, we demonstrate that these observations can be explained by ^1^H → ^13^C CCR during sample transfer.

**Figure 5:**
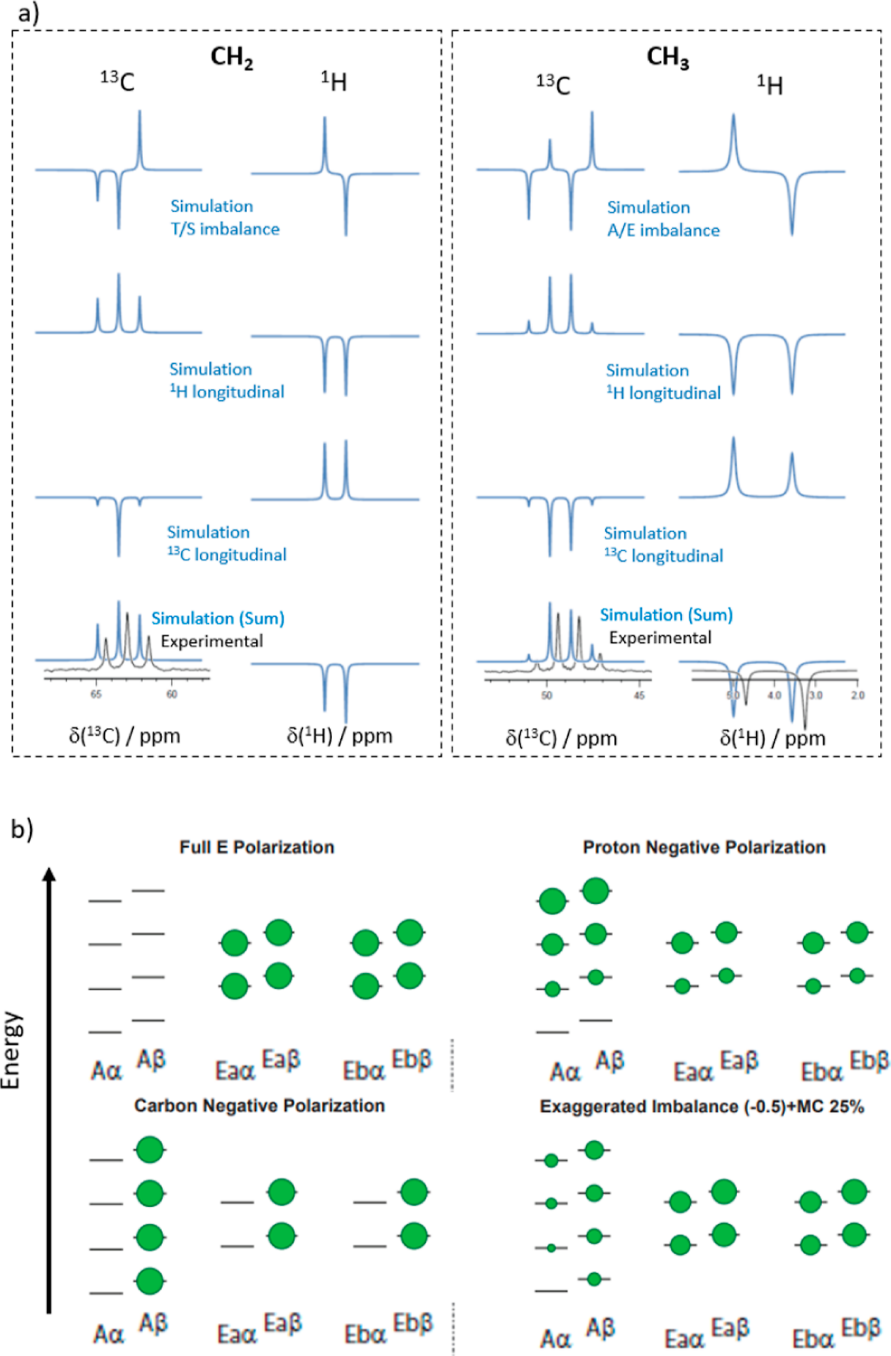
Contributions of different spin states to the experimentally detected ^13^C spectrum (corresponding to *t* = 0 in Figures 6 and 7) due to CCR. Both CH_2_ and CH_3_ groups could be simulated by combining longitudinal ^1^H_z_ and ^13^C_z_ magnetizations with population imbalances between the respective irreducible representations (T–S and A–E, respectively). The former two contributions lead to roughly symmetric multiplets, while the population imbalances lead to asymmetric multiplets. Their superposition agrees with the observed signals. Note that the indicated magnetization types correspond to population distributions produced in the solid via DNP, while the detected multiplets stem from superpositions of in-phase and anti-phase coherences after dissolution. (b) Sketch of the population distributions for the methyl group used to simulate the data in panel (a). The sizes of the green spheres are proportional to the populations of the energy levels. The bottom right panel shows a combination of an A–E imbalance (with overpopulated E-states), negative ^1^H_Z_ polarization, and negative ^13^C_Z_ polarization. For visibility, the contributions of the A–E imbalance and the ^13^C_Z_ polarization have been exaggerated. The representation of the states used in the simulations can be found in the Supporting Information.

Figure 5a shows the positive signal intensities of the methanol ^13^CH_3_ quadruplet after negative DNP and transfer to the NMR spectrometer for detection. After injection (at *t* = 0), the four lines of the ^13^CH_3_ quadruplet have been fitted to intensity ratios of (+0.8:+3.3:+3.1:+1.0) ± 0.08 that depart from the usual +1:+3:+3:+1 ratios observed in the thermal equilibrium. This can be understood when assuming that the spin state of a methyl group immediately after dissolution, that is, before passage through the low-field region, comprises a linear combination of different distributions of populations. Three types were found important: ^1^H_Z_ longitudinal magnetization of all three methyl protons, ^13^C_Z_ longitudinal magnetization, and an A–E imbalance.

Although our theoretical considerations outlined above show that the influence of A–E imbalances on CCR is relatively weak compared to that of longitudinal magnetization, such population imbalances are often encountered in DDNP experiments involving methyl groups.^14,21,38−41^ The overpopulation of either the lowest (after positive DNP) or highest (after negative DNP) energy levels in the solid state before dissolution necessarily leads to a population imbalance between the A and E symmetry manifolds. E-state overpopulation is expected after negative DNP of methyl groups undergoing rapid proton tunneling as the latter results in an energy increase of the E-states relative to that of the A-states. A detailed explanation can be found in refs (21) and (42). A-state overpopulation is expected for all cases of positive DNP and negative DNP in slowly tunneling systems.^38^Figure 5b illustrates the population differences between the various spin levels for the A–E imbalance, the longitudinal ^1^H_Z_ magnetization, and ^13^C_Z_ magnetization, as well as a typical linear combination. Note that such initial populations are expected for DNP using the nitroxide TEMPOL since this polarization agent can polarize both protons and carbon-13 nuclei simultaneously, albeit with lesser efficiency for the latter, unless cross-polarization techniques are used.^43^

Many experiments have been performed using pyruvate without observing the effect reported in this work. This can be ascribed to the fact that such experiments used narrow-band radicals such as trityls to hyperpolarize the carbon-13 spins in the context of clinical or preclinical hyperpolarized MRI.^24,27^ Since these radicals do not hyperpolarize proton spins efficiently, the phenomenon described here is not expected in these experiments. However, as other types of radicals that polarize both proton and carbon spins are more common for applications in physical chemistry,^44,45^ we expect more reports of such an effect in this context.

### NMR Signal Phase

To model the signal amplitudes and their evolution as a function of time after dissolution, we employed the SpinDynamica^35^ software package and let the spin system evolve under the total relaxation superoperator, including CSA, DD, DD–CSA, and random field relaxation. The Supporting Information contains detailed codes and information about these simulations. We could reproduce the experimental signal intensities when considering an initial distribution ^1^H_z_/^13^C_z_/A–E = −1:–0.01:3 directly after dissolution (at *t* = −12 s). This combination of populations is transformed into a distribution with an opposite sign for ^13^C_z_ at *t* = 0 s.

Figure 5a shows how the different components of the identified population combination contribute to the experimentally observed signal at the time of detection, that is, after the transfer during which CCR acts. While the A–E and T–S imbalances lead to a ^13^C signal in the anti-phase with respect to the ^1^H spins and to the inversion of two lines, relaxation of longitudinal ^1^H_z_ magnetization leads to an inversion of all four lines, and longitudinal ^13^C_z_ magnetization leads to the expected non-inverted line shape. Only combining all three contributions enabled us to reproduce the experimentally observed line shape, an inverted multiplet with asymmetric line intensities.

Note that the magnetization types indicated in Figure 5 correspond to the magnetization produced in a solid via DNP, while the detected multiplets stem from superpositions of in-phase and anti-phase contributions. Indeed, CCR of the three produced magnetization types creates C_z_, C_z_H_z_^(1)^, C_z_H_z_^(1)^H_z_^(2)^, and C_z_H_z_^(1)^H_z_^(2)^H_z_^(3)^ type spin states, which upon applying a detection pulse on the carbon channel lead to anti-phase and in-phase coherences.

**Figure 6:**
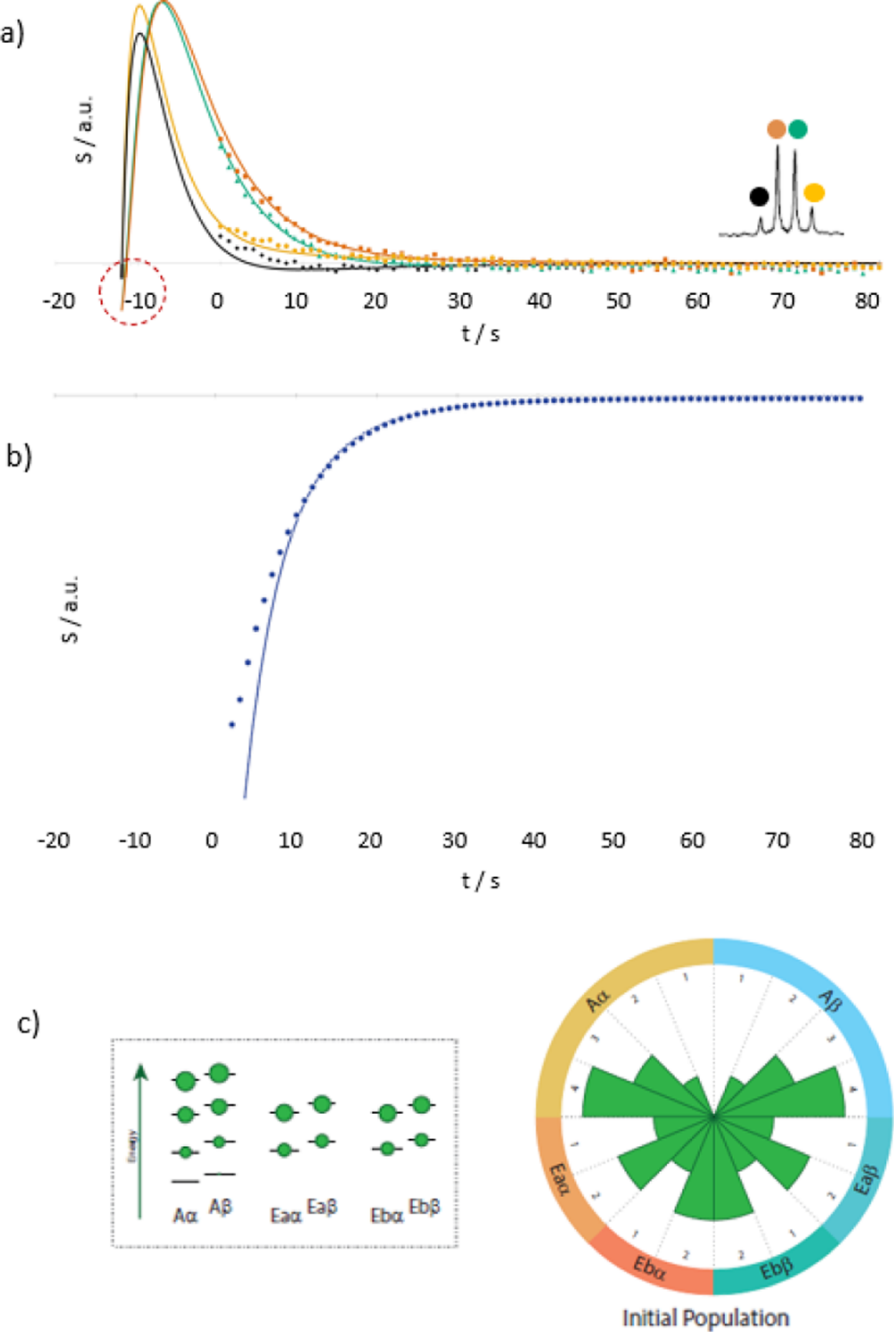
(a) ^13^C decay curves of hyperpolarized methanol after injection into the NMR spectrometer (dotted curves). The color code indicates the different lines of the methanol quartet, as indicated in the inset. The solid lines stem from a simulation using a starting polarization consisting of an A–E imbalance and longitudinal proton and longitudinal carbon magnetizations, as described in the main text. NMR observation started at time point *t* = 0. Extrapolations of populations before NMR detection are expressed by *t* < 0. (b) Mono-exponential decay (dotted line) of the ^1^H signal of ^12^CH_3_ in natural abundance methanol detected in parallel to the ^13^CH_3_ decay curves shown in panel (a). (c) Sketch of the population distributions of the methyl group used to simulate the data in panels (a,b). The sizes of the green spheres are proportional to the populations of the energy levels. The right panel visualizes the populations in the circular representation described in the Theory section.

In Figure 6a, it can be seen that the aforementioned population distribution enabled simulations of line amplitudes for the ^13^C quartet that match the experimentally observed ones well throughout the entire detection period (*i.e.*, for *t* > 0). In other words, not only the static spectrum at the time of injection but also its evolution could be reproduced. We used a static magnetic field of 11.7 T for the simulations and a relaxation superperator, as shown in Figure 4. All other details on the simulation (coupling constants, Hamiltonians, *etc.*) can be found in the Supporting Information. Note that the signal detected experimentally upon the arrival of the dissolved, hyperpolarized sample in the NMR spectrometer corresponds to *t* = 0 s in the simulations in Figure 6a. In other words, the simulations assumed an initial distribution at *t* = −12 s such that *t* = 0 coincides with the onset of the experimental NMR detection. In this manner, the simulations reproduce the time evolutions of the experimental signal amplitudes.

The theoretical considerations in Figures 3 and 4 already showed that DD CCR involving both carbon and proton spins is responsible for the inversion of the signal. This can be corroborated by our simulations. When the DD relaxation mechanism is removed from the relaxation superoperator, the inversion of magnetization is canceled (Figures S1–S3 of the Supporting Information). Canceling any other relaxation mechanism changes the computed line shapes and time dependence, but the signal inversion remains. Hence, it can be inferred that cross-correlated relaxation effects, in particular DD CCR, lead to an inversion of the ^13^C_z_ magnetization, which converts the A–E imbalance and the positive longitudinal ^1^H magnetization into negative ^13^C_z_ polarization.

In other words, by selectively discarding various contributions from the relaxation superoperator, we found that DD CH–CH relaxation is responsible for converting the largest share of ^1^H_Z_ magnetization into ^13^C_Z_ magnetization (Figures S1–S3 of the Supporting Information). Werbelow and Grant already reported the influence of nonequilibrium ^1^H_Z_ magnetization on ^13^C_Z_ magnetization in their seminal work on relaxation.^46^ However, the effects were relatively weak as only thermal equilibrium magnetization was involved, in stark contrast to the hyperpolarized case at hand. It should be noted that CSA relaxation can also invert smaller amounts of the ^13^C_Z_ order, although this contribution is expected to decrease with the magnetic field (or even to be negligible in ZULF NMR^47^).

Complementarily, Figure 6b shows the mono-exponential behavior (dotted line) of the (positive) observed ^1^H_Z_ magnetization, confirming the high ^1^H polarization underlying our simulations. The data stem mostly from ^12^CH_3_ methyl groups since the sample had natural isotopic ^13^C abundance. However, simulations for ^13^CH_3_ methyl groups (solid line) obtained using the same parameters as those used to fit the ^13^C data result in a biexponential behavior of the (*negative*) ^1^H magnetization. Figure 6c represents the population distributions used for the simulation.

**Figure 7:**
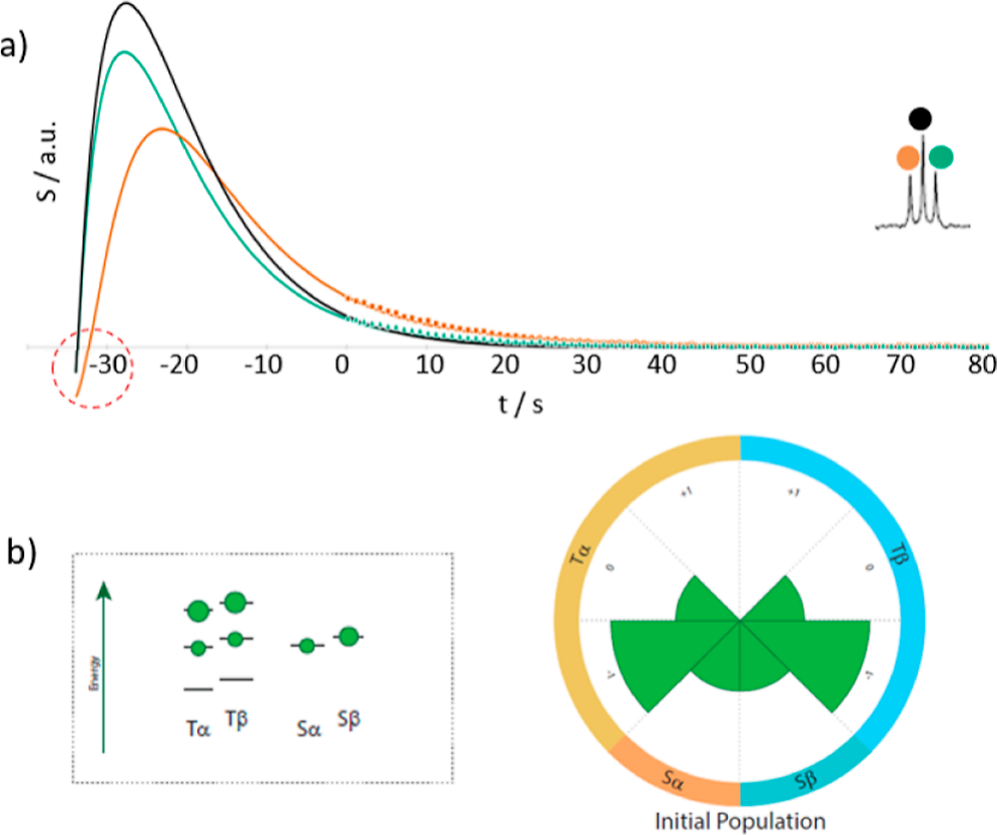
Build-up and decay curves of ^13^CH_2_ groups in hyperpolarized EG after transfer to the NMR spectrometer (dotted curves). The color code indicates the three lines of the ^13^CH_2_ triplet, as indicated in the inset. The solid lines stem from a simulation using a starting polarization consisting of a triplet–singlet imbalance T–S and longitudinal proton and longitudinal carbon magnetizations, as described in the main text. NMR observation started at time point *t* = 0. Extrapolations to the time before NMR detection are represented by *t* < 0. (b) Sketch of the population distributions of the methyl group used to simulate the data in panel (a). The sizes of the green spheres are proportional to the populations of the energy levels. The right panel visualizes the populations in the circular representation described in the Theory section.

For the ^13^CH_2_ signals of EG, similar simulations as for the methyl groups could reproduce the experimental observations. Only the A–E imbalance of CH_3_ groups had to be replaced by the triplet–singlet (T–S) imbalance of the CH_2_ groups.^22^ Hence, the behavior of both methyl and methylene groups can be explained equivalently by CCR effects. For methylene, a starting magnetization ^1^H_Z_/^13^C_Z_/T–S = 1:0.01:0.5 was found to provide a good match between simulated and experimental data (Figure 7).

It should be noted that the signals of deuterated glycerol are not inverted for two reasons. First, the hyperpolarization levels achieved for the deuterons are not as high as those achieved for protons and decay much faster under quadrupolar relaxation.^39,40^ Second, the CCR processes leading to signal inversion observed for protons are not efficient in the case of deuterons.^35,36^ In contrast, the signal of the quaternary ^13^C nucleus in pyruvate–C1 is not inverted as all neighboring nuclei to the detected ^13^C are largely NMR inactive.

### Rate of Relaxation

In our simulations, relatively long intervals of *t* = 12 and 34 s between the start of the trajectory and the time point when the system reaches the intensity ratio of the observed multiplets have been assumed to describe the methyl and methylene groups, respectively. However, in our DDNP experiments, the sample transfer took only 5 s. The passage through low magnetic fields was much shorter. Hence, relaxation processes must become faster during sample transfer to account for our observations. However, semi-classical calculations using RedKite^36^ did not show a strong field dependence on the relaxation rates in an isolated methyl group.

In contrast, to reproduce the experimentally observed behavior, the influence of free radicals in the solution during sample transfer had to be considered. Indeed, Jannin and co-workers have shown^48^ that ^13^C relaxation in the presence of TEMPOL radicals is significantly accelerated at low magnetic fields. More importantly, for the present context, Kiryutin and co-workers^31^ have shown that ^1^H relaxation rates accelerate by orders of magnitude due to the low-field passage in the presence of TEMPOL. Hence, the reduced time needed for the signal inversion of the protonated species might be accounted for by faster paramagnetically induced relaxation during sample transfer in our DDNP experiments. This assumption is supported by the studies of Ghose and Prestegard,^49^ Boisbouvier *et al.*,^50^ Bertini *et al.*,^51,52^ and Madhu *et al.*(53) These authors showed that interference between paramagnetic relaxation enhancement and heteronuclear DD relaxation impacts the relaxation properties of paramagnetic systems by enhancing DD relaxation and creating multi-spin order.^53^ The latter can lead to magnetization transfers between different nuclei. Importantly, this effect is strongly field-dependent.^53^

**Figure 8:**
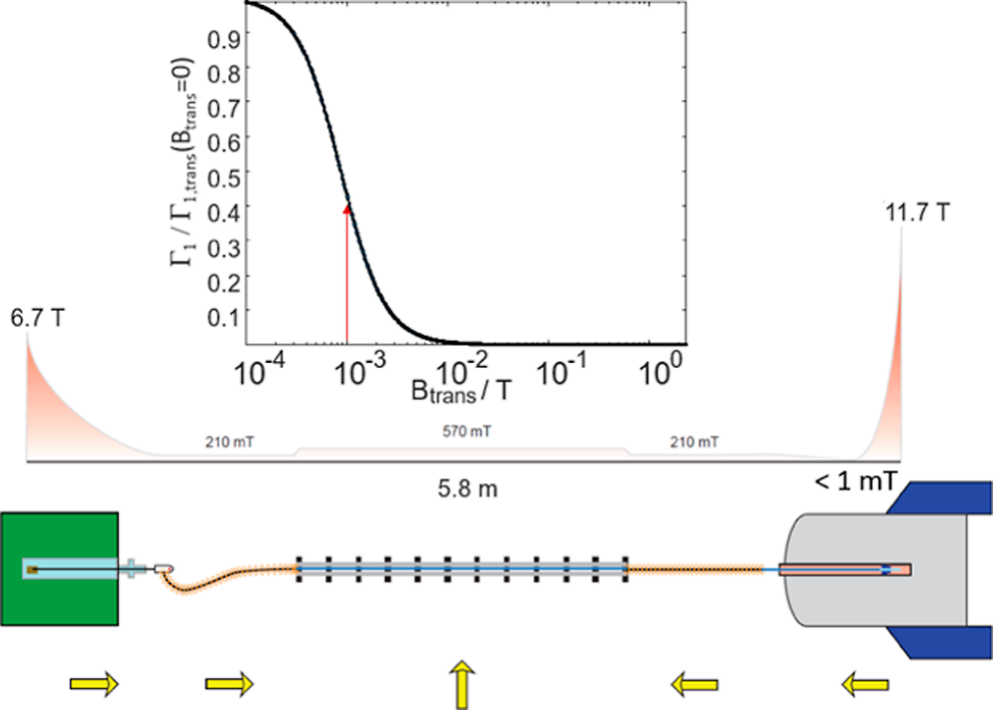
Sketch of the magnetic fields encountered during sample transport. The yellow arrows mark the direction of the fields. Within the magnetic tunnel, a field of *B*_0_ > 570 mT is achieved using a four-element Halbach array. Between the polarizer/NMR spectrometer and the tunnel, pulsed solenoids produced a magnetic field of *ca. B*_0_ = 210 mT. Between the solenoid outlet and the Bruker “Ultra-Shielded” NMR spectrometer, the field dropped to *B*_0_ < 1 mT. The inset shows how the longitudinal paramagnetic relaxation enhancement due to the 0.36 mM TEMPOL in the solution is accelerated due to the low-field passage (see the main text). Indeed, at a field of 1 mT, the relaxation rate increases rapidly compared to the field of 11.7 T within the NMR spectrometer.

A simulation of the field dependence of solvent paramagnetic relaxation enhancements (sPREs) further supports that strong relaxation enhancements can be induced by codissolved radicals. Figure 8 shows a sketch of the different magnetic fields encountered during the transfer in the Vienna laboratory (see also Figure S4). Between the outlet of the tunnel and the entrance to the NMR spectrometer bore, the transient magnetic field drops to less than 1 mT. The inset shows how the longitudinal proton relaxation rate due to dissolved TEMPOL Γ_1_ (black curve) changes with the magnetic field. The curve was calculated following the spectral density function for the sPRE proposed by Okuno and co-workers.^54^ Details on the calculations can be found in the Supporting Information. The red arrow indicates the transiently encountered magnetic field of 1 mT. The sPRE dramatically boosts relaxation rates during the low-field passage. Therefore, the low-field passage likely increases relaxation rates significantly at magnetic fields encountered during the sample transfer. Hence, the discrepancy between 5 s experimental transfer times and the >10 s theoretically calculated delays can possibly be accounted for by paramagnetically accelerated DD relaxation during sample transfer.

## Conclusions

In conclusion, we show that different relaxation mechanisms taking place during sample transfer can drastically affect the outcome of DDNP experiments. NMR spectra can be inverted, and hyperpolarized spin states can be effectively depleted or unexpectedly enhanced.

These effects are amplified by so-called “ultra-shielded” NMR magnets that provide only minimal stray fields, which can accelerate relaxation between hyperpolarized protons and carbon spins when paramagnetic polarization agents remain in the solution after dissolution. To avoid the effect of cross-relaxation in ^13^C NMR spectra, one may employ deuterated molecules. Alternatively, a magnetic tunnel that guides the sample into the bore of the NMR spectrometer can slow down relaxation effects.^30^

Besides, we show how simulations and “backpropagation” of the experimental time dependence can be employed to determine the state of the spin system back to the moment of dissolution.

The growing popularity of DDNP in combination with novel magnet shielding technologies will likely lead to more observations of signal inversion effects, and the presented contribution can help identify and quantify these phenomena.

## Supporting Information Available

Simulation of CH_3_ group relaxation using neglected mechanisms, simulation of CH_3_ group relaxation with different A–E imbalance, representation of the population used for the simulation, ^1^H T_1_ relaxation with various field variations, simulation parameters, SpinDynamica code for CH_3_ and CH_2_ group relaxation, and calculation of the sPRE (PDF)

## Notes

All data and codes are available at DOI: 10.5281/zenodo.6580927.
