# Supplementary material for: Inversion of Hyperpolarized 13C NMR Signals through Cross-Correlated Cross-Relaxation in Dissolution DNP Experiments
Source: J Phys Chem B. 2022 Jun 8;126(24):4599–610. doi: 10.1021/acs.jpcb.2c03375 (PMC9234958; doi:10.1021/acs.jpcb.2c03375)
Supplement: Supplementary file 1 — jp2c03375_si_001.pdf [file jp2c03375_si_001.pdf]

Supporting Information for 'Inversion of Hyperpolarized ^{13}C NMR Signals Through Cross-Correlated Cross-Relaxation in Dissolution DNP Experiments'

Mattia Negroni[1], David Guarin[2,3], Kateryna Che[1], Ludovica M. Epasto[1], Ertan Turhan[1], Albina Selimović[1], Fanny Kozak[1], Samuel Cousin[4], Daniel Abergel[5], Geoffrey Bodenhausen[5], Dennis Kurzbach[1,]*

[1] University Vienna, Faculty of Chemistry, Institute of Biological Chemistry, Währinger Str. 38, 1090 Vienna, Austria

[2] Athinoula A. Martinos Center for Biomedical Imaging, Department of Radiology, Massachusetts General Hospital, Charlestown, Massachusetts 02129, USA

[3] Polarize ApS, 1808 Frederiksberg, Denmark

[4] Institut de Chimie Radicalaire - UMR 7273, Saint-Jérôme Campus, Av. Esc. Normandie Niemen, Aix-Marseille Université / CNRS, 13397 Marseille, Cedex 20, France

[5] Laboratoire des Biomolécules, LBM, Département de chimie, École Normale Supérieure, PSL University, Sorbonne Université, CNRS, 24 rue Lhomond, 75005 Paris, France

** E-mail: dennis.kurzbach@univie.ac.at*

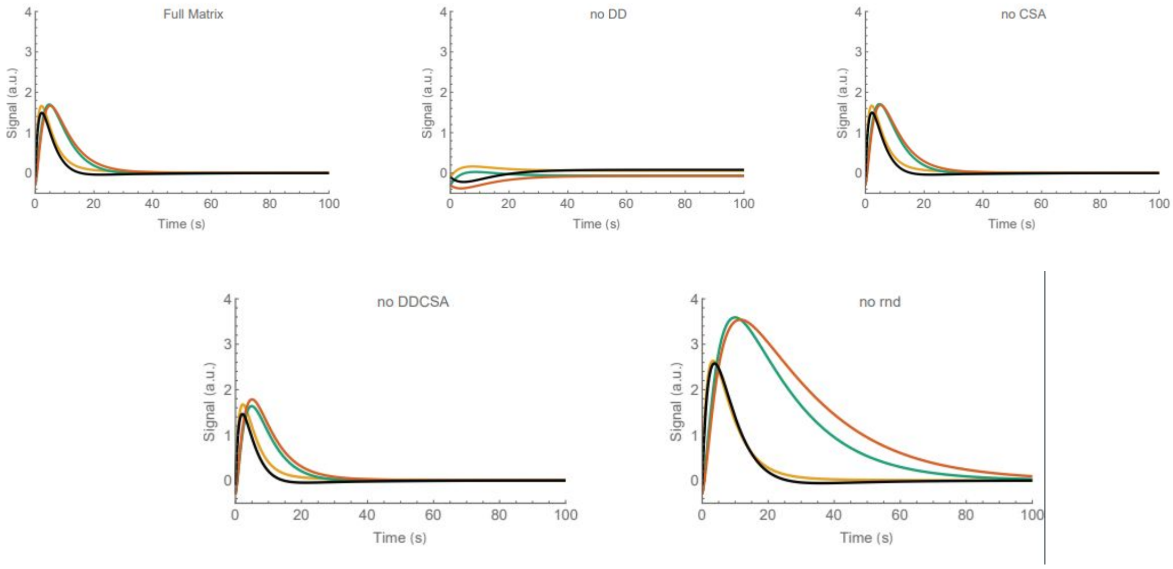

Figure S1. Simulations of methyl group relaxation starting from -1:-0.01:3 (^1H_Z:$^{13}C_Z$:A-E) order for individual carbon transitions. As indicated in each panel, different relaxation mechanisms were neglected in the simulations. Obviously, a lack of DD relaxation leads to only very weak transfer from the ^{1}H nuclei to the ^{13}C nucleus.

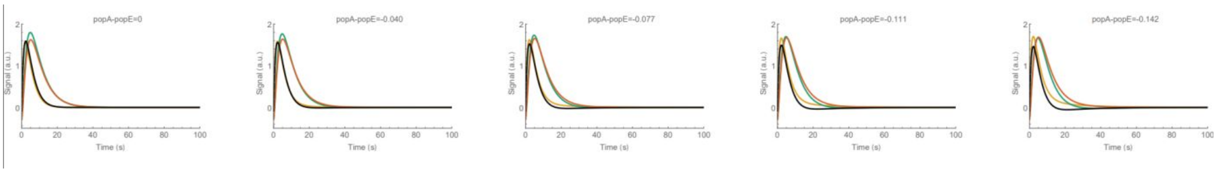

Figure S2. Influence of the A-E imbalance on the ^{1}H-^{13}C CCR starting from -1:-0.01:x (^1H_Z:$^{13}C_Z$:A-E), the value for x is shown at the top of each panel.

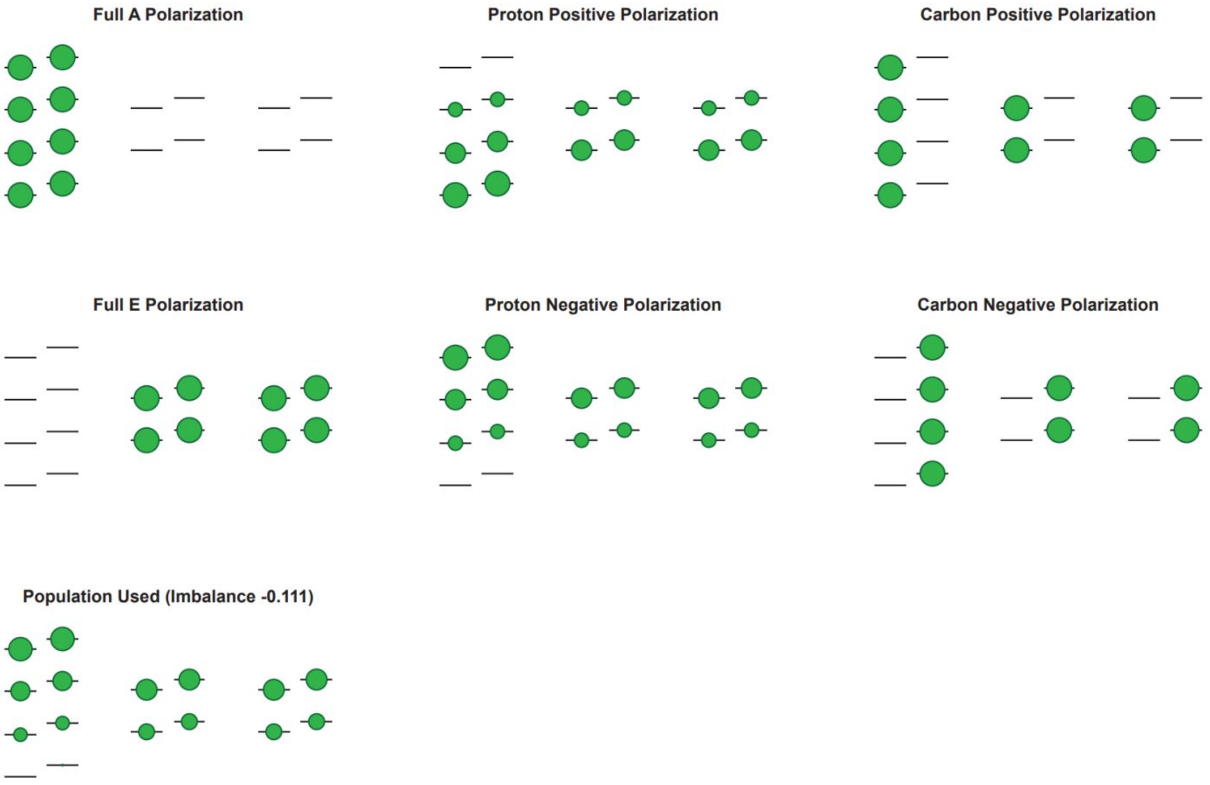

Figure S3. Representation of the polarization used to simulate the methyl ^{13}C time traces in the main text.

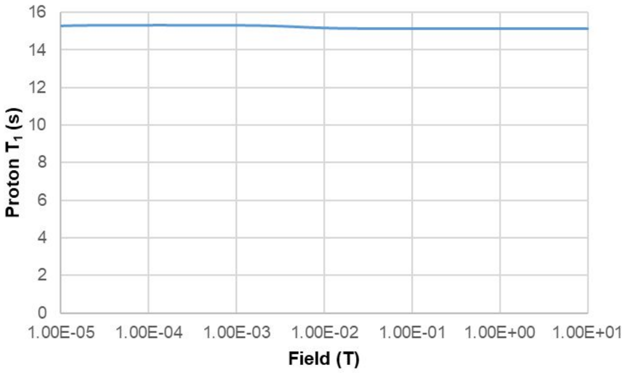

Figure S4. Simulated methyl ^{1}H longitudinal relaxation time at various magnetic fields switches starting from 6,7 T and considering DD, CSA, DDCSA and rnd mechanisms, but no paramagnetic relaxation enhancement.

Simulations of CH_3 and CH_2 Relaxation.

The signal evolutions (main text Fig. 3 and 4, SI Fig. S1 and S2) were simulated using a modified version of SpinDynamica notebook of reference [1] and runs with Mathematica 12.0 and SpinDynamica 3.5.0.

The relaxation superoperator Γ was generated using Redfield's theory of relaxation by dipolar coupling (DD), chemical shift anisotropy (CSA) and random field fluctuations (rnd):

$$\Gamma = \Gamma_{DD-DD} + \Gamma_{CSA-CSA} + \Gamma_{DD-CSA} + \Gamma_{rnd-rnd} \qquad \text{(S1)}$$

For the first three terms we modeled the systems as rigid rotors using the following formula:

$$\Gamma_{\ldots - \ldots} = \sum_{\iota,\iota'} \xi_\iota \xi_{\iota'} \sum_{m=-2}^{2} \frac{\tau_C \tau_R}{2\tau_R + m^2 \tau_C} D^2_{0,m}(\Omega_\iota) D^2_{0,m}(\Omega_{\iota'}) \sum_{m=-2}^{2} (-1)^m \left[T^m_{\iota,2}, T^{-m}_{\iota',2}\right] \qquad \text{(S2)}$$

Where ι and ι′ indices refer either to the atoms involved in case of CSA, or the pair of atoms in case of DD interaction, τ_C is the correlation time, τ_R is the rotation time, D is a Wigner-D matrix element, Ω_ι are the angle between the interaction and the molecular frame, T are the irreducible spherical tensors, and ξ are the interaction coefficients. Note that the DD-CSA term in eq. S1 accounts for DD-CSA cross-correlated cross relaxation, while the DD-DD and CSA-CSA terms account for both cross-correlated as well as auto-relaxation.

The term accounting for random field fluctuations is given by:

$$\Gamma_{rnd-rnd} = \sum_{\iota,\iota'} - Br_\iota Br_{\iota'} \tau_C \sum_{m=-1}^{1} (-1)^m \left[T^m_{\iota,1}, T^{-m}_{\iota',1}\right] \qquad \text{(S3)}$$

Where Br indicates the statistical random field fluctuation at the position of the spin. We considered all the fluctuation correlated for each couple ι ι′. We considered a fluctuation of 30000π 1/s for the proton and 22000π 1/s for carbons.

The relaxation behavior of the spin system was then computed by solving the master equation in Liouville space (see Bengs et al.[2]):

$$\frac{d}{dt}\rho(t) = \widehat{\mathscr{L}}(t)\rho(t)$$

Where ρ is the density matrix and L the Liouvillian.

The time traces of Fig. S2 were calculated by selectively removing a term from eq. S1.

The simulation parameter used are:

	τ_C	τ_R	δ CSA ^{1}H	δ CSA ^{13}C
CH_3	14 ps	1,4 ps	5 ppm	34 ppm
CH_2	5.0 ps	2.0 ps	6 ppm	60 ppm

where δ is the anisotropy parameter according to the Haeberlen-Mehring-Spiess formalism.[3]

To solve the master equation, the relaxation superoperator was combined with a time-independent Hamiltonian to create the Liouville superoperator, as suggested by Dumez et al.[1]

Dipolar interactions were calculated using a CH bond length of 110 pm and assuming tetrahedral configurations around the central carbons. All further details can be found in the commented simulation codes below.

SpinDynamica Code for CH_3 group

This code was adapted from Dumez et al. [1] Details about the spin physics behind the simulations can be found therein and in the associated Supporting Information. For the code used here, comments are shown in green, headlines in blue. The comments and headlines guide the reader step-by-step through the code.

```
Needs["SpinDynamica`"]
```

Setting System

```
SetSpinSystem[{{1,1/2},{2,1/2},{3,1/2},{4,1/2}}]; (* 4 Spin ½ particles *)
IsotopicType[1|2|3] = 1; (* 3 Protons *)
IsotopicType[4] = 13; (* 1 Carbon nucleus *)
```

Geometry

```
rCH = 1 1.1 10^-10; (* CH bond length in m *)
aCH = 1*70.5Degree; (* CH bind angle time 0.5 *)
hCH = rCH*Cos[aCH];
hHH = 3/2 Sqrt[rCH^2-hCH^2];
rHH = hHH/Sin[60 Degree];
coordinates = {{-(1/3)hHH,1/2 rHH,hCH},{-(1/3)hHH,-(1/2)rHH,hCH},{2/3 hHH,0,hCH},{0,0,0}}; (* atom coordiantes *)
RR[j_,k_]:=Distance[coordinates[[j]],coordinates[[k]]] (* atom-to-atom distances *)
```

Hamiltonian

```
Hparams := {ωCSH-> 2 Pi 0,ωCSC-> 2 Pi 0,JCH-> 127,JHH-> 0} (* Hamiltonian parameters, rotating frame Larmor frequencies and J-coupling constants *)
HCS = ωCSH Sum[opI[i,"z"],{i,1,3}]+ωCSC opI[4,"z"]; (* nuclear Zeeman *)
HJCH = 2 Pi JCH opI[4,"z"]. opI[{1,2,3},"z"]; (* CH J-coupling *)
HJHH = 2 Pi JHH (opI[1].opI[2]+opI[1].opI[3]+opI[2].opI[3]); (* HH J-coupling *)
H = HCS+HJCH+HJHH; (* total Hamiltonian *)
```

Basis Set

```
SetBasis[ZeemanBasis[]];
```

A symmetry basis Kets (* Setup Basis Kets in the symmetrized Basis, irreducible representation A *)

```
KetsAα={BasisKets[][[1]],Sqrt[1/3](BasisKets[][[2]]+BasisKets[][[3]]+BasisKets[][[5]]),Sqrt[1/3](BasisKets[][[7]]+BasisKets[][[6]]+BasisKets[][[4]]),BasisKets[][[8]]};
KetsAβ={BasisKets[][[9]],Sqrt[1/3](BasisKets[][[10]]+BasisKets[][[11]]+BasisKets[][[13]]),Sqrt[1/3](BasisKets[][[15]]+BasisKets[][[14]]+BasisKets[][[12]]),BasisKets[][[16]]};
```

E symmetry basis Kets (* Setup Basis Kets in the symmetrized Basis, irreducible representation E *)

```
KetsEaα={Sqrt[1/3](BasisKets[][[5]]+Exp[-I 2π/3]BasisKets[][[2]]+Exp[I 2π/3]BasisKets[][[3]]),-Sqrt[(1/3)](BasisKets[][[4]]+Exp[-I 2π/3]BasisKets[][[7]]+Exp[I 2π/3]BasisKets[][[6]])};
KetsEaβ={Sqrt[1/3](BasisKets[][[13]]+Exp[-I 2π/3]BasisKets[][[10]]+Exp[I 2π/3]BasisKets[][[11]]),-Sqrt[(1/3)](BasisKets[][[12]]+Exp[-I 2π/3]BasisKets[][[15]]+Exp[I 2π/3]BasisKets[][[14]])};
KetsEbα={Sqrt[1/3](BasisKets[][[5]]+Exp[I 2π/3]BasisKets[][[2]]+Exp[-I 2π/3]BasisKets[][[3]]),-Sqrt[(1/3)](BasisKets[][[4]]+Exp[I 2π/3]BasisKets[][[7]]+Exp[-I 2π/3]BasisKets[][[6]])};
KetsEbβ={Sqrt[1/3](BasisKets[][[13]]+Exp[I 2π/3]BasisKets[][[10]]+Exp[-I 2π/3]BasisKets[][[11]]),-Sqrt[(1/3)](BasisKets[][[12]]+Exp[I 2π/3]BasisKets[][[15]]+Exp[-I 2π/3]BasisKets[][[14]])};
```

Symmetrized basis Kets (* Combine A and E *)

SymmetrizedBasisKets=Join[KetsAα,KetsAβ,KetsEaα,KetsEaβ,KetsEbα,KetsEbβ];
DefineBasis[SymmetrizedBasis,SymmetrizedBasisKets,CheckBasis->True];

Relaxation Superoperator

SetOperatorBasis[ShiftAndZOperatorBasis[]]

Interactions

ω0H = LarmorFrequency[1,B0]; (* compute proton Larmor Frequency *)
ω0C=LarmorFrequency[13,B0]; (* compute carbon Larmor Frequency *)
ωDD[i_,j_]:=DirectDipolarCoupling[IsotopicType[i],IsotopicType[j],RR[i,j]]; (* Dipolar Coupling *)

ΩDDPR[j_,k_]:=AxesToEuler[AxisSystem[coordinates[[k]]-coordinates[[j]]],AxisSystem[-ez]] (* Angles for Wigner Matrix *)
ωCSA[i_]:=LarmorFrequency[IsotopicType[i],B0]*δCSA[i] 10^-6; (* CSA contribution *)
ΩCSAPR[4]:={0,0,0}; (* Coordinate system centered around 13C *)
ΩCSAPR[i_]:=ΩDDPR[i,4];

DD relaxation

ΓDDterm[{i_,j_},{k_,l_}]:=-(6/5)ωDD[i,j]ωDD[k,l](Sum[(τC τR)/(τR + m1^2 τC) WignerD[2,{0,m1}][ΩDDPR[i,j]]Conjugate[WignerD[2,{0,m1}][ΩDDPR[k,l]]],{m1,-2,2}]Sum[(-1)^mDoubleCommutationSuperoperator[opT[{i,j},{2,m}],opT[{k,l},{2,-m}]],{m,-2,2}]) (* Solving the double commutator formalism *)
ΓDD = Superoperator[Sum[ΓDDterm[{i,j},{k,l}],{i,1,3},{j,i+1,4},{k,1,3},{l,k+1,4}]]; (* Sum up relaxation superoperator for dipolar relaxation *)

CSA relaxation

constCSA = Sqrt[3/8];
opTCSA[i_,m_]:=NullOperator[];
opTCSA[i_,0]:=Sqrt[8/3]opI[i,"z"]; (* Iz operators *)
opTCSA[i_,1]:=-opI[i,"+"]; (* positive ladder operators *)
opTCSA[i_,-1]:=opI[i,"-"]; (* negative ladder operators *)
ΓCSAterm[i_,k_]:=-(1/5) constCSA^2 ωCSA[i]ωCSA[k](Sum[(τC τR)/(τR + m1^2 τC) WignerD[2,{0,m1}][ΩCSAPR[i]]Conjugate[WignerD[2,{0,m1}][ΩCSAPR[k]]],{m1,-2,2}] Sum[(-1)^m DoubleCommutationSuperoperator[opTCSA[i,m],opTCSA[k,-m]],{m,-1,1}]) (* Solving the double commutator formalism *)
ΓCSA = Superoperator[Sum[ΓCSAterm[i,j],{i,1,4},{j,1,4}]]; (* Sum up relaxation superoperator for CSA relaxation *)

DD/CSA relaxation

ΓDDCSAterm[{i_,j_},k_]:=-((constCSA Sqrt[6])/5)ωDD[i,j]ωCSA[k](Sum[(τC τR)/(τR + m1^2 τC) WignerD[2,{0,m1}][ΩDDPR[i,j]]Conjugate[WignerD[2,{0,m1}][ΩCSAPR[k]]],{m1,-2,2}]Sum[(-1)^mDoubleCommutationSuperoperator[opT[{i,j},{2,m}],opTCSA[k,-m]],{m,-1,1}]+Sum[(τC τR)/(τR + m1^2 τC) WignerD[2,{0,m1}][ΩCSAPR[k]]Conjugate[WignerD[2,{0,m1}][ΩDDPR[i,j]]],{m1,-2,2}]Sum[(-1)^mDoubleCommutationSuperoperator[opTCSA[k,m],opT[{i,j},{2,-m}]],{m,-1,1}]) (* Solving the double commutator formalism *)

ΓDDCSA = Superoperator[Sum[ΓDDCSAterm[{i,j},k],{i,1,3},{j,i+1,4},{k,1,4}]]; (* Sum up relaxation superoperator for DD/CSA relaxation *)

Correlated random-field relaxation

Br[1|2|3]=BrH; (* random field fluctuations experiences by protons *)

Br[4]=BrC; (* random field fluctuations experiences by the carbon nucleus *)

Γrandterm[i_,j_] := -τC Br[i]Br[j]Sum[(-1)^m DoubleCommutationSuperoperator[opT[i,{1,m}],opT[j,{1,-m}]],{m,-1,1}]; (* Solving the double commutator formalism *)

Γrand= Superoperator[Sum[Γrandterm[i,j],{i,1,4},{j,1,4}]]; (* Sum up relaxation superoperator for random field relaxation *)

Total relaxation superoperator

Γtot = Superoperator[ΓDD+ΓDDCSA+ΓCSA+Γrand]; (* sum up total relaxation superoperator *)

Γtotsec=Secularize[Γtot,{{1,2,3},{4}}]; (* secularize relaxation superoperator *)

Initial state and observables

A initial state

SpinThreeHalvesInitialState=Operator[SparseArray@DiagonalMatrix[Join[Table[1/8,{8}],Table[0,{8}]]],SymmetrizedBasis]; (* spin state for a "perfect" A-E imbalance with overpopulated A states *)

Single-transition operators, 13C

opSTA1z = SingleTransitionOperator[{{1,5}d ,SymmetrizedBasis},"z"]; (* compute single transition operator between levels 1 and 5 in the symmetrized basis, see above *)

opSTA2z = SingleTransitionOperator[{{2,6},SymmetrizedBasis},"z"];

opSTA3z = SingleTransitionOperator[{{3,7},SymmetrizedBasis},"z"];

opSTA4z = SingleTransitionOperator[{{4,8},SymmetrizedBasis},"z"];

opSTEa1z = SingleTransitionOperator[{{9,11},SymmetrizedBasis},"z"];

opSTEa2z = SingleTransitionOperator[{{10,12},SymmetrizedBasis},"z"];

opSTEb1z = SingleTransitionOperator[{{13,15},SymmetrizedBasis},"z"];

opSTEb2z = SingleTransitionOperator[{{14,16},SymmetrizedBasis},"z"];

Single-transition operators , 1H

opST1H1=opI[{1,2,3},"z"].opI[4,"α"];

opST1H2=opI[{1,2,3},"z"].opI[4,"β"];

Trajectories

```
peak1op = opSTA1z; (* Observable for peak 1  of the methyl quartet*)
peak2op=1/3 (opSTA2z+opSTEa1z+opSTEb1z); (* Observable for peak 2  of the methyl quartet*)
peak3op=1/3 (opSTA3z+opSTEa2z+opSTEb2z); (* Observable for peak 3  of the methyl quartet*)
peak4op=opSTA4z; (* Observable for peak 4 of the methyl quartet*)
relaxparams ={τC-> 1.4 10^-11, τR-> 0.14 10^-11,B0->11,7,δCSA[1|2|3]->5,δCSA[4]->34,BrH-> 2 Pi 15 10^3,BrC-> 2 Pi 11 10^3,κHH-> 1,κCH-> 1}; (* relaxation parameters for all four mechanisms *)
Lsop=CombineGenerators[Γtotsec/.relaxparams,H/.Hparams]; (* combine Hamiltonian and relaxation superoperator to create the Liouvillian *)
dLsop2 = NDiagonalize[Lsop]; (* diagonalize Liouvillian *)

{peak1fun,peak2fun,peak3fun,peak4fun,ST1H1fun,ST2H2fun}=Trajectory[SpinThreeHalvesInitialState+opI[1,"z"]+opI[2,"z"]+opI[3,"z"]+0.01*opI[4,"z"])->{peak1op,peak2op,peak3op,peak4op,opST1H1,opST1H2},{dLsop2,400}]; (* Compute evolution of the spin system using the SpinDynamica routine *)
```

13C plot

```
Plot[{10Re@peak1fun[t],10Re@peak2fun[t],10Re@peak3fun[t],10Re@peak4fun[t]},{t,0,400},PlotRange->Full] (* now plot *)
```

1H plot

```
Plot[{Re@ST1H1fun[t],Re@ST2H2fun[t]},{t,0,400},PlotRange->Full] (* now plot *)
```

Spin Dynamica Code for CH2 group

```
Needs["SpinDynamica`"]
```

Setting System

```
SetSpinSystem[{{1,1/2},{2,1/2},{3,1/2}}]; (* 3 Spin ½ particles *)
IsotopicType[1|2] = 1; (* 2 Protons *)
IsotopicType[3] = 13; (* 1 Carbon nucleus *)
```

Geometry

```
rCH = 1 1.1 10^-10; (* CH bond length in m *)
aCH = 1*70.5Degree; (* CH bind angle time 0.5 *)
coordinates = {{rCH Cos[aCH],rCH Sin[aCH],0},{rCH Cos[aCH],-rCH Sin[aCH],0},{0,0,0}}; (* atom coordiantes *)
RR[j_,k_]:=Distance[coordinates[[j]],coordinates[[k]]] (* atom-to-atom distances *)
```

Hamiltonian

```
Hparams:={ωCSH-> 2 Pi 0,ωCSC-> 2 Pi 0,JCH-> 127,JHH-> 0}(* Hamiltonian parameters, rotating frame Larmor frequencies and J-coupling constants *)
HCS=ωCSH Sum[opI[i,"z"],{i,1,2}]+ωCSC opI[3,"z"];(* nuclear Zeeman *)
HJCH = 2 Pi JCH opI[3,"z"]. opI[{1,2},"z"];(* CH J-coupling *)
HJHH = 2 Pi JHH (opI[1].opI[2]); (* HH J-coupling *)
H = HCS+HJCH+HJHH; (* total Hamiltonian *)
```

Basis Set

```
SetBasis[ZeemanBasis[]];
```

T basis Kets (* Setup Basis Kets in the symmetrized Basis, triplet states *)

```
KetsTα={BasisKets[][[1]],Sqrt[1/2](BasisKets[][[3]]+BasisKets[][[2]]),BasisKets[][[4]]};
KetsTβ={BasisKets[][[5]],Sqrt[1/2](BasisKets[][[7]]+BasisKets[][[6]]),BasisKets[][[8]]};
```

S basis Kets (* Setup Basis Kets in the symmetrized Basis, singlet states *)

```
KetsSα={Sqrt[1/2](BasisKets[][[3]]-BasisKets[][[2]])};
KetsSβ={Sqrt[1/2](BasisKets[][[7]]-BasisKets[][[6]])};
```

Symmetrized basis Kets

```
SymmetrizedBasisKets=Join[KetsTα,KetsTβ,KetsSα,KetsSβ]; (* Combine T and S *)
```

Relaxation Superoperator

```
SetOperatorBasis[ShiftAndZOperatorBasis[]]
```

Interactions

```
ω0H = LarmorFrequency[1,B0]; (* compute proton Larmor Frequency *)
ω0C=LarmorFrequency[13,B0]; (* compute carbon Larmor Frequency *)
ωDD[i_,j_]:=DirectDipolarCoupling[IsotopicType[i],IsotopicType[j],RR[i,j]]; (* Dipolar Coupling *)
ΩDDPR[j_,k_]:=AxesToEuler[AxisSystem[coordinates[[k]]-coordinates[[j]]],AxisSystem[-ez]] (* Angles for Wigner Matrix *)
ωCSA[i_]:=LarmorFrequency[IsotopicType[i],B0]*δCSA[i] 10^-6; (* CSA contribution *)
ΩCSAPR[3]:={0,0,0}; (* Coordinate system centered around 13C *)
ΩCSAPR[i_]:=ΩDDPR[i,3];
```

DD relaxation

```
ΓDDterm[{i_,j_},{k_,l_}]:=-(6/5)ωDD[i,j]ωDD[k,l](Sum[(τC τR)/(τR + m1^2 τC) WignerD[2,{0,m1}][ΩDDPR[i,j]]Conjugate[WignerD[2,{0,m1}][ΩDDPR[k,l]]],{m1,-2,2}]Sum[(-1)^mDoubleCommutationSuperoperator[opT[{i,j},{2,m}],opT[{k,l},{2,-m}]],{m,-2,2}]) (* Solving the double commutator formalism *)
```

ΓDD = Superoperator[Sum[ΓDDterm[{i,j},{k,l}],{i,1,2},{j,i+1,3},{k,1,2},{l,k+1,3}]];(* Sum up relaxation superoperator for dipolar relaxation *)

CSA relaxation

```
constCSA = Sqrt[3/8];
opTCSA[i_,m_]:=NullOperator[];
opTCSA[i_,0]:=Sqrt[8/3]opI[i,"z"]; (* Iz operators *)
opTCSA[i_,1]:=-opI[i,"+"]; (* positive ladder operators *)
opTCSA[i_,-1]:=opI[i,"-"]; (* negative ladder operators *)
ΓCSAterm[i_,k_]:=-(1/5) constCSA^2 ωCSA[i]ωCSA[k](Sum[(τC τR)/(τR + m1^2 τC) WignerD[2,{0,m1}][ΩCSAPR[i]]Conjugate[WignerD[2,{0,m1}][ΩCSAPR[k]]],{m1,-2,2}]Sum[(-1)^m DoubleCommutationSuperoperator[opTCSA[i,m],opTCSA[k,-m]],{m,-1,1}])(* Solving the double commutator formalism *)
ΓCSA = Superoperator[Sum[ΓCSAterm[i,j],{i,1,3},{j,1,3}]];(* Sum up relaxation superoperator for CSA relaxation *)
```

DD/CSA relaxation

```
ΓDDCSAterm[{i_,j_},k_]:=-((constCSA Sqrt[6])/5)ωDD[i,j]ωCSA[k](Sum[(τC τR)/(τR + m1^2 τC) WignerD[2,{0,m1}][ΩDDPR[i,j]]Conjugate[WignerD[2,{0,m1}][ΩCSAPR[k]]],{m1,-2,2}]Sum[(-1)^mDoubleCommutationSuperoperator[opT[{i,j},{2,m}],opTCSA[k,-m]],{m,-1,1}]+Sum[(τC τR)/(τR + m1^2 τC) WignerD[2,{0,m1}][ΩCSAPR[k]]Conjugate[WignerD[2,{0,m1}][ΩDDPR[i,j]]],{m1,-2,2}]Sum[(-1)^mDoubleCommutationSuperoperator[opTCSA[k,m],opT[{i,j},{2,-m}]],{m,-1,1}])(* Solving the double commutator formalism *)
ΓDDCSA = Superoperator[Sum[ΓDDCSAterm[{i,j},k],{i,1,2},{j,i+1,3},{k,1,3}]];(* Sum up relaxation superoperator for DD/CSA relaxation *)
```

Correlated random-field relaxation

```
Br[1|2]=BrH; (* random field fluctuations experiences by protons *)
Br[3]=BrC; (* random field fluctuations experiences by the carbon nucleus *)
Γrandterm[i_,j_] := -τC Br[i]Br[j]Sum[(-1)^m DoubleCommutationSuperoperator[opT[i,{1,m}],opT[j,{1,-m}]],{m,-1,1}];(* Solving the double commutator formalism *)
Γrand= Superoperator[Sum[Γrandterm[i,j],{i,1,3},{j,1,3}]];(* Sum up relaxation superoperator for random field relaxation *)
```

Total relaxation superoperator

```
Γtot = Superoperator[ΓDD+ΓDDCSA+ΓCSA+Γrand]; (* sum up total relaxation superoperator *)
Γtotsec=Secularize[Γtot,{{1,2},{3}}](* secularize relaxation superoperator *)
```

Initial state and observables

T initial state

SpinInitialState=Operator[SparseArray@DiagonalMatrix[Join[Table[1/6,{6}],Table[0,{2}]]],SymmetrizedBasis]; ; (* spin state for a “perfect” T-S imbalance with overpopulated T states *)

Single-transition operators, 13C

opSTT1z = SingleTransitionOperator[{{1,4},SymmetrizedBasis},"z"]; (* compute single transition operator between levels 1 and 4 in the symmetrized basis, see above *)

opSTT2z = SingleTransitionOperator[{{2,5},SymmetrizedBasis},"z"];
opSTT3z = SingleTransitionOperator[{{3,6},SymmetrizedBasis},"z"];
opSTSz = SingleTransitionOperator[{{7,8},SymmetrizedBasis},"z"];

Single-transition operators , 1H

opST1H1=opI[{1,2},"z"].opI[3,"α"];
opST1H2=opI[{1,2},"z"].opI[3,"β"];

Trajectories

peak1op = opSTT1z; (* Observable for peak 1 of the methylene triplet*)
peak2op=1/2 (opSTT2z+opSTSz); (* Observable for peak 2 of the methylene triplet*)
peak3op=opSTT3z; (* Observable for peak 3 of the methylene triplet*)

relaxparams ={τC-> 5.0 10^-12, τR-> 2.0 10^-12,B0->11.7,δCSA[1|2|3]->6,δCSA[4]->60,BrH-> 2 Pi 15 10^3,BrC-> 2 Pi 11 10^3};(* relaxation parameters for all four mechanisms *)
Lsop=CombineGenerators[Γtotsec/.relaxparams,H/.Hparams]; (* combine Hamiltonian and relaxation superoperator to create the Liouvillian *)
dLsop = NDiagonalize[Lsop]; (* diagonalize Liouvillian *)
{peak1fun,peak2fun,peak3fun,ST1H1fun,ST2H2fun}=Trajectory[-(0.5*SpinInitialState+opI[1,"z"]+opI[2,"z"]+0.01*opI[3,"z"])->{peak1op,peak2op,peak3op,opST1H1,opST1H2},{dLsop,200}]; (* Compute evolution of the spin system using the SpinDynamica routine *)

13C plot

Plot[{1000Re@peak1fun[t],1000Re@peak2fun[t],1000Re@peak3fun[t]},{t,0,100},PlotRange->Full] (* now plot *)

1H plot

Plot[{1000Re@ST1H1fun[t],1000Re@ST2H2fun[t]},{t,0,100}](* now plot *)

Solvent Paramagnetic Relaxation Enhancement (sPRE) Calculations.

The calculations (main text Fig. 7) were carried out as outlined in reference [4]. In brief, the longitudinal sPRE rate Γ_1 was calculated as

$$\Gamma_1 = \frac{2}{15} K^2 S(S+1) J_{approx}(\omega) \qquad \text{(S4a)}$$

$$K = \frac{\mu_0}{4\pi} \gamma \beta_m g \qquad \text{(S4b)}$$

Where γ is the gyromagnetic ratio, g the g-factor, S the electron spin, ω the nuclear Larmor frequency, β_m the Bohr magneton, μ_0 the vacuum permeability, and $J_{approx}(\omega)$ the spectral density function:

$$J_{approx}(\omega) = \frac{J(0)}{1 + a\omega^2 + b\sqrt{\omega}} \qquad \text{(S5)}$$

$$\langle r^{-6} \rangle_{\text{norm}} = \frac{J(0)}{n_S \tau_C} \qquad \text{(S6)}$$

Where *r* is the average distance between the electrons and the nucleus, n_s the electron concentration (0.36 mM) and τ_c the correlation time. For the calculations shown in the main text, the parameters *a* and *b* where chosen to be 7 10^{-10} and 6 10^{-6}, to match the results of reference.[2] τ_c was set to 1 10^{-12} s and the average distance <*r*> to 20 nm. However, it should be noted that <*r*> and τ_c are correlated parameters and that various combinations can lead to similar results. Hence, the values given here have to be considered with caution.

References

1. Dumez, J. N.; Hakansson, P.; Mamone, S.; Meier, B.; Stevanato, G.; Hill-Cousins, J. T.; Roy, S. S.; Brown, R. C.; Pileio, G.; et al, Theory of long-lived nuclear spin states in methyl groups and quantum-rotor induced polarisation. *J Chem Phys* **2015,** *142* (4), 044506.
2. Bengs, C.; Levitt, M. H., SpinDynamica: Symbolic and numerical magnetic resonance in a Mathematica environment. *Magn Reson Chem* **2017,** 56(6): 374–414.
3. Schmidt-Rohr, K.; Spiess, H. W., *Multidimensional Solid-Sate NMR and Polymers*. Academic Press: San Diego, CA, 1996.
4. Okuno, Y.; Szabo, A.; Clore, G. M., Quantitative Interpretation of Solvent Paramagnetic Relaxation for Probing Protein-Cosolute Interactions. *J Am Chem Soc* **2020,** *142* (18), 8281-8290.
